# Neue Wege in der Hörrehabilitation nach Cochleaimplantation

**DOI:** 10.1007/s00106-020-00914-0

**Published:** 2020-08-27

**Authors:** C. Völter, C. Schirmer, M. Röber, D. Hinsen, S. Dazert, K. Bilda

**Affiliations:** 1 Klinik für Hals‑, Nasen- und Ohrenheilkunde, Kopf- und Halschirurgie der Ruhr-Universität Bochum, Katholisches Klinikum Bochum gGmbh, Bleichstr. 15, 44787 Bochum, Deutschland; 2 Kampmann Hörsysteme, Bochum, Deutschland; 3 Hochschule für Gesundheit (hsg), Bochum, Deutschland

**Keywords:** Hörrehabilitation, Digitalisierung, Computerbasiertes Hörtraining, Telerehabilitation, Aural rehabilitation, Digitalization, Computer-based auditory training, Telerehabilitation

## Abstract

**Einleitung:**

Nach einer Cochleaimplantation (CI) ist i.d.R. ein Hör- und Sprachtraining erforderlich, das bislang entweder ambulant oder stationär im direkten Kontakt mit einem Therapeuten erfolgt. Bedingt durch technische Weiterentwicklungen, aber vor allem durch die zunehmende Nutzung digitaler Medien und dem Wunsch nach Mobilität und Selbständigkeit steigt der Bedarf an digitalen Trainingsprogrammen auch im Rahmen der Hörrehabilitation.

**Material und Methoden:**

In einem ersten Schritt wurden entsprechend den gängigen Hör- und Sprachverarbeitungsmodellen die wichtigsten Elemente eines Hörtrainings definiert und die Kernbausteine für eine computerbasierte teletherapeutische Hörplattform bestimmt. Dabei wurden lerntherapeutisch orientierte Elemente der Motivationsförderung sowie Mechanismen der Adaptivität, wie sie im Rahmen der logopädischen Therapie eingesetzt werden, berücksichtigt. In einem zweiten Schritt erfolgte die Entwicklung eines ersten Prototyps der teletherapeutischen Hörplattform „train2hear“ im Rahmen eines interdisziplinären Forschungsprojektes.

**Ergebnisse:**

Kernbaustein des vorgestellten „train2hear“ Hörtrainings ist eine an die „International Classification of Functioning, Disability, and Health“ (ICF) angelehnte Eingangsanalyse, aufgrund derer ein individueller Trainingsplan erstellt wird. Verschiedene Adaptivitätsmechanismen dienen dazu, den Schwierigkeitsgrad kontinuierlich an den individuellen Lernfortschritt des Nutzers anzupassen. Ein teletherapeutisches Videotool ermöglicht den Austausch mit dem Therapeuten vor Ort.

**Diskussion/Fazit:**

Die vorgestellte Plattform „train2hear“ stellt einen ersten deutschsprachigen Prototyp einer computerbasierten Hörrehabilitation dar. Dieser muss nun im Rahmen einer klinischen Studie im Hinblick auf die Anwendbarkeit und Effektivität evaluiert und weiterentwickelt werden.

## Abstract (en)

**Introduction:**

After cochlear implantation, auditory and speech training is usually necessary. Up until now, this has been performed on an out- or inpatient basis in direct contact with a therapist. Due to technical advances but also particularly because of an increasing use of digital media and a desire for mobility and independence, the demand for digital training programs is increasing in the field of hearing rehabilitation.

**Materials and Methods:**

In a first step, the most important elements of auditory training were determined on the basis of commonly used auditory and speech processing models, and the core features of a computer-based teletherapeutic hearing platform were defined. This process considered motivational elements and adaptive mechanisms as regularly used in educational and speech and language therapy. In a second step, an initial prototype of the “train2hear” teletherapeutic hearing platform was developed by an interdisciplinary research team.

**Results:**

The core of the train2hear platform is an initial analysis according to the International Classification of Functioning, Disability, and Health (ICF), on the basis of which an individual training plan is designed. Various adaptive mechanisms enable the level of difficulty to be continually adapted to the individual userʼs learning progress. A videoconference tool enables contact between the patient and the therapist.

**Conclusion:**

The train2hear platform represents a first prototype of a computer-based auditory rehabilitation program in German. Further evaluation and development in a clinical study are needed to determine the feasibility and efficacy of the platform.

Die Anzahl schwerhöriger Menschen nimmt – bedingt durch die demographische Entwicklung – rasant zu. Damit verbunden steigt auch die Anzahl an Cochleaimplantationen und derer, die sich in einer hörtherapeutischen Rehabilitation befinden. Bislang findet diese überwiegend stationär oder ambulant im direkten Kontakt mit einem Therapeuten statt. Digitale Hörtrainingsprogramme und computerbasierte teletherapeutische Hörplattformen bieten hier neue Chancen der Rehabilitation. Vorgestellt werden soll ein erster Prototyp für erwachsene Cochlea-Implantat(CI)-Träger.

## Einleitung

Die Versorgung mit einem Cochlea-Implantat (CI) ist für viele hochgradig schwerhörige beziehungsweise taube Patienten die einzige Möglichkeit, (wieder) besser hören und Sprache verstehen zu können. Mit 466 Mio. Betroffenen weltweit ist Schwerhörigkeit eine der häufigsten chronischen Erkrankungen, von der vor allem über 65-Jährige stark betroffen sind [23, 47]. Bedingt durch die demographische Entwicklung, aber auch durch eine Ausweitung des Indikationsspektrums hat die Anzahl der Cochleaimplantationen in den vergangenen Jahrzehnten rasant zugenommen. So waren allein 2015 in Deutschland ca. 25.000–30.000 Personen mit einem oder zwei CIs versorgt [10]. Entscheidend für einen optimalen Sprachgewinn ist die postoperative Hörrehabilitation, die individuell auf den einzelnen CI-Träger abgestimmt sein sollte [1]. Diese findet derzeit in Deutschland meist im Einzelsetting, zum Teil auch in der Gruppe im Rahmen einer Face-to-face-Therapie in einem spezialisierten Rehabilitationszentrum stationär oder ambulant statt [1, 19]. Im Hinblick auf den zunehmenden Fachkräftemangel in der Hör‑, Sprach- und Sprechtherapie sind hier alternative Versorgungsmodelle erforderlich.

Nicht nur die Anzahl der mit einem CI versorgten Patienten, sondern auch die technischen Möglichkeiten in der Rehabilitation haben im Zeitalter der Digitalisierung einen enormen Zuwachs erhalten. Das Gesundheitswesen erfährt aktuell zum einen durch das e‑Health-Gesetz und die digitale Agenda, aber auch durch das deutlich steigende digitale Medieninteresse der Bevölkerung einen Wandel [37]. Die Entwicklung von und das Interesse an digitalen Lösungen, die der Prävention und Vermeidung von Krankheiten ebenso wie der therapeutischen oder rehabilitativen Begleitung dienen, nehmen dabei stark zu [4, 31]. Da diese Programme große Datenmengen sammeln und verarbeiten können, ist hierdurch neben einer Steigerung der Effektivität und Effizienz eine stärkere Individualisierung des Trainings möglich [35]. Gleichzeitig wächst die Nutzung digitaler Produkte und Infrastrukturen, wie von mobilen Daten, Tablets und Smartphones, auch im höheren Alter. So betrug 2017 der Anteil der Smartphone-Nutzer über 65 Jahren bereits 41 % mit steigender Tendenz [42].

Diese technischen Entwicklungen bieten neue Möglichkeiten, auch im Rahmen der Hörrehabilitation nach einer Cochleaimplantation. In der neurologischen Rehabilitation finden sich bereits Konzepte, die eine computerbasierte Rehabilitation enthalten [8, 11, 14, 28]. Erste Untersuchungen, so unter anderem von Choi et al., konnten über eine hohe Zufriedenheit von Aphasikern mit einer computerbasierten Therapie und eine signifikante Verbesserung ihrer sprachlichen Funktionen im Follow-up berichten [8]. Ähnlich positive Erfahrungen beschrieben auch Macoir et al., welche die kommunikativen Funktionen von 20 Aphasiepatienten nach einem 3‑wöchigen Training über die Plattform Oralys Tele Therapy untersuchten [28]. Dabei scheint ein teletherapeutisches Training einer Face-to-face-Therapie, wie Dial et al. nachwiesen, im Hinblick auf den Therapieerfolg vergleichbar zu sein [11]. Auch im Bereich der Stottertherapie gibt es Studien über den effektiven Einsatz einer Teletherapie, so unter anderem von Euler, der nach einer Teletherapie ähnlich gute Erfolge im Hinblick auf die Verbesserung der Stotterrate aufzeigen konnte wie in der Präsenzgruppe [14].

Im Hinblick auf die Hörrehabilitation gibt es derzeit nur eine überschaubare Anzahl computer- und internetbasierter Hörtrainingsprogramme, meist auf Hörgerätetragende ausgerichtet. Diese dienen vor allem dazu, die Gewöhnung an den neuen Höreindruck mit der Hörhilfe zu unterstützen [44]. Viele der Programme sind käuflich zu erwerben, teilweise nur in Kombination mit entsprechenden Übungshörgeräten oder durch einen lizensierten Akustiker (z. B. Renova®, Fa. Hörzentrum Böhler GmbH, Augsburg; Sensoton®, Fa. Hoertraining-online.de, Hamburg; Hörpilot®, Fa. Deppermann, Palsbröcker, Rosenstengel GbR, Bielefeld; terzo® Gehörtherapie, Fa. ISMA AG, Sonneberg). Daneben finden sich computerbasierte Hörtrainingsprogramme, die speziell auf CI-Träger ausgerichtet sind [44]. Diese weisen jedoch derzeit noch Lücken auf, vor allem bezogen auf die Adaptivität, Individualität und die Komplexität der Übungen [44] und sie werden bislang meist nur als Zusatzangebot zur konventionellen Reha eingesetzt. Eine Implementierung digitaler Hörtrainingsprogramme im Rahmen der Hörrehabilitation könnte langfristig eine kostengünstige und ressourcenschonende Alternative darstellen, mit einem hohen Maß an räumlicher und zeitlicher Flexibilität bei gleichzeitig verbesserter Effizienz und Effektivität [35].

Ziel des vorliegenden Forschungsprojektes war es daher, einen ausgereiften Prototyp einer computerbasierten Hörtrainingsplattform („train2hear“) für erwachsene CI-Träger zu entwickeln.

## Material und Methoden

Gängige Hör- und Sprachverarbeitungsmodelle und Konzepte der Hörtherapie wurden mittels einer Literaturrecherche ermittelt und in Bezug auf ihre Relevanz für die Rehabilitation nach Cochleaimplantation untersucht. Die entsprechenden Methoden und Merkmale wurden gesammelt und in der theoretischen Entwicklung der Hörplattform berücksichtigt. In einem zweiten Schritt wurden hierauf aufbauend die zentralen Elemente eines Hörtrainings für erwachsene CI-Träger definiert und gemeinsam mit den Softwareentwicklern in das theoretische Grundgerüst der Plattform integriert. Daneben wurden verschiedene Theorien zur Motivationssteigerung im Hinblick auf (digitales) Lernen recherchiert und an eine computerbasierte Hörtherapie angepasst. Anschließend erfolgten die Entwicklung einer computerbasierten Hörplattform und die Programmierung einer ersten Prototypversion. Um eine hohe Nutzerfreundlichkeit und -orientierung zu gewährleisten, wurden die potenziellen Nutzer im Rahmen von Anwenderworkshops während des gesamten Entwicklungsprozesses einbezogen.

## Ergebnisse

### Theoretische Grundlage der Hörrehabilitation

Eine Hörrehabilitation sollte sich mit dem Ziel der Optimierung des auditiven Sprachverstehens am individuellen Prozess der Hörentwicklung orientieren und eine kontinuierliche Anpassung an den individuellen Leistungsstand ermöglichen [12, 36]. Basis für die Wiederherstellung der Sprachwahrnehmung und des Sprachverstehens sind die vier hierarchisch aufeinander aufbauenden Entwicklungsstufen des Hörens, und zwar die Detektion, die Diskrimination, die Identifikation und das Verstehen [13]. Darüber hinaus beschreiben verschiedene Hör- und Sprachverarbeitungsmodelle die Verarbeitung vom auditorischen Input bis zur Verarbeitung auf Wortebene bei Normalhörenden [40]. Während serielle Modelle, wie das Kohortenmodell [18, 29], den Ablauf der einzelnen Verarbeitungskomponenten in einer festen Reihenfolge wiedergeben, erfolgt nach konnektionistischen Modellen, wie dem TRACE-Modell, die Informationsverarbeitung gleichzeitig analytisch, d. h. „bottom up“, und/oder synthetisch, d. h. „top down“ [30]. Daneben spielen neurokognitive Fähigkeiten, vor allem das Arbeitsgedächtnis, eine Rolle für das Sprachverstehen im Störlärm und damit auch für die Hörrehabilitation, wie Rönnberg et al. in ihrem ELU(„ease of language understandig“)-Modell näher ausgeführt haben [38]. Das Motivationskonzept orientiert sich an der „Self Determination Theory“ von Ryan und Deci, welche die Selbstbestimmung, das Bedürfnis nach Kompetenz und Wirksamkeit sowie die soziale Eingebundenheit und Zugehörigkeit als Schlüsselelemente beschreiben [39].

### Ziele der Hörplattform

Folgende Ziele wurden für die Erstellung der Hörplattform herausgearbeitet: So sollte das Hörtraining zwar patientenorientiert, aber therapeutengeführt sein und sowohl analytische als auch synthetische Elemente enthalten. Der Aufbau der Übungen sollte strukturiert [25] und stufenartig unter Berücksichtigung der Ausgangsbedingungen, aber auch der individuellen Ziele konzipiert sein und sich im Trainingsverlauf selbstständig adaptiv an den Trainingsfortschritt anpassen können [12]. Des Weiteren sollte eine möglichst hohe Adhärenz durch eine abwechslungsreiche Gestaltung, aber auch ein detailliertes positives Feedback erreicht werden. Als vorrangiges Ziel des Hörtrainings wurde eine Verbesserung des Sprachverstehens im Alltag [24, 36], insbesondere bei Vorhandensein von Hintergrundgeräuschen, definiert.

### Entwicklung einer Hörplattform

In einem interdisziplinären Team, bestehend aus in der Hörtherapie und -forschung tätigen Therapeuten, Softwareentwicklern und Audiologen, wurde die angestrebte Hörtrainingsplattform zunächst in der Theorie geplant. Besonderen Einfluss in diesen Entwicklungsprozess hatten zum einen die oben beschriebenen theoretischen Grundlagen der Hörrehabilitation inklusive einer Recherche nach bisher verfügbaren Programmen. Diese ergab, dass sich die zu entwickelnde Software u. a. durch die Erhebung von personenbezogenen Daten und Umweltfaktoren sowie Informationen zur aktuellen Hörfähigkeit und ebenso durch eine automatische adaptive Anpassung des Programms an den Lernfortschritt des Patienten von den bisherigen Programmen unterscheiden soll. Zum anderen wurde der Bedarf an digitalen Hörtrainingsprogrammen durch eine Bedarfsanalyse an schwerhörigen erwachsenen Personen erhoben. Demzufolge sollten aus Benutzerperspektive alltagsnahe Hörübungen mit abwechslungsreichen Materialien und Störgeräuschen in die Entwicklung einfließen.

### Aufbau der Hörplattform

**Figure 1:**
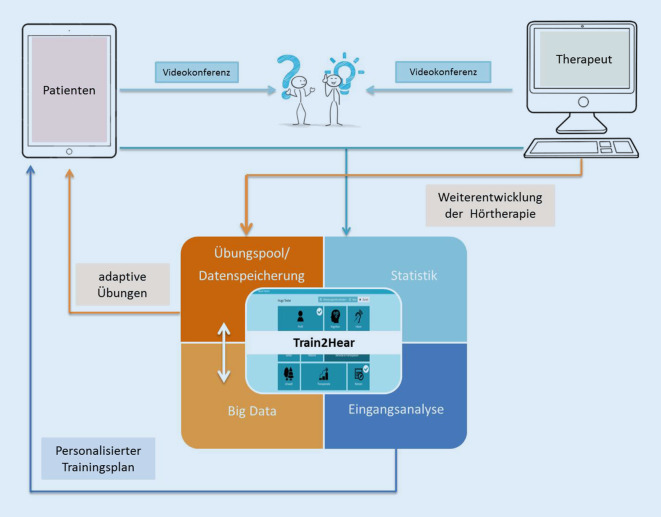

Anschließend wurde entsprechend den zuvor erwähnten Kriterien eine teletherapeutische Übungsplattform gestaltet. Insgesamt wurden drei Benutzeroberflächen eingerichtet, eine für die Nutzung durch die Patienten, eine zweite für Interventionen durch die Therapeuten und eine dritte für administrative Anwendungen, die das gesamte Sprachmaterial verwalten und die gesammelten Daten der Patienten analysieren und speichern (Abb. 1). Entwickelt wurde das Programm für das Betriebssystem iOS. Ausgerichtet auf Tablets steht es ausschließlich auf iPads zur Verfügung. Um das Programm als CI-Träger nutzen zu können, muss zuvor eine Freischaltung durch einen berechtigten Therapeuten erfolgen. Dieser ist in der Lage, nach einer Eingangsanalyse einen Patienten anzulegen.

#### Patientenplattform

Das Patienten-Back-End ist in drei Bereiche gegliedert. Zentrales Element ist der individuelle Trainingsplan. Darüber hinaus gibt es einen freien Trainingsbereich und die Möglichkeit, mit einem Therapeuten eine Videokonferenz zur Beratung oder aber zur Durchführung einzelner teletherapeutischer Übungen durchzuführen.

#### Therapeutenplattform

Im Therapeuten-Back-End erfolgen das Anlegen und Freischalten eines Patienten für das Übungsprogramm. Zusätzlich hat der Therapeut Einblick in den Trainingsverlauf der Patienten und kann über eine Videokonferenz mit dem Patienten kommunizieren.

#### Administrative Plattform

Das Programm enthält momentan ca. 500 Einzelwörter sowie ca. 600 Sätze unterschiedlicher Länge und 50 Texte. Daneben kann das Programm auf 500 Minimalpaare und 300 Silben von männlichen und weiblichen Sprechern sowie auf 50 Geräusche (inklusive Instrumente) und 25 alltagsnahe Störgeräusche, wie z. B. Straßenverkehrslärm, Restaurantkulisse, Geräusche im Zug und Stimmengewirr (in einer SNR [signal-to-noise-ratio] von −20 bis +20 dB [Dezibel]), zugreifen. Die Auswahl des Sprachmaterials, beziehungsweise der Geräusche, erfolgt zufällig durch den Computer, jedoch passend zum Thema der Aufgabe, um unerwünschte Listenlerneffekte zu verhindern. Darüber hinaus werden im administrativen Back-End sämtliche Übungsdaten gespeichert und analysiert, so im Hinblick auf die Anzahl oder Art der Fehler ebenso wie die Häufigkeit und Art der Inanspruchnahme von Hilfeleistungen oder die Dauer pro Übung. Diese Daten können nicht nur vom Therapeuten eingesehen werden, sondern werden auch in verkürzter Form für den Patienten einsehbar grafisch dargestellt.

### Bausteine der Hörplattform

#### Eingangsanalyse

Zentraler Baustein zu Beginn des Hörtrainings ist die *Eingangsanalyse*, die in Anlehnung an die ICF (International Classification of Functioning, Disability, and Health) die persönlichen fördernden und hemmenden Einflussfaktoren und die Umweltfaktoren erfasst und untergliedert ist in die Teilbereiche Profil, Kognition, Hören, Sehen, Motorik, Aktivität und Partizipation, Umwelt und Therapieziele (Abb. 1). Im Bereich des Profils erhebt der Therapeut persönliche Angaben des Hörgeschädigten, wie beispielsweise Vor- und Zunamen, Geburtsdatum, E‑Mail-Adresse und Geschlecht, und fragt auch nach einer Berufstätigkeit und den privaten Lebensumständen. An neurokognitiven Fähigkeiten werden neben dem Arbeitsgedächtnis das Kurz- und Langzeitgedächtnis, die Verarbeitungsgeschwindigkeit, die Aufmerksamkeit und die Inhibition zuvor mithilfe einer computerbasierten Testbatterie eruiert [45, 46] und anschließend in die Eingangsanalyse eingegeben. Daneben werden das Sprachverstehen in Ruhe mit dem Freiburger Zahlen- und Einsilbertest sowie im Störlärm mit dem Oldenburger Satztest erfasst.

Weiterhin werden die persönlichen Ziele des Patienten bzw. Nutzers im Hinblick auf das Hören und Sprachverstehen mit dem Implantat festgehalten. Ebenso werden die Teilbereiche Sehen und Motorik, die sich auf die Bedienung des Programms auswirken könnten, erhoben. Unter der Kategorie Aktivität und Partizipation werden im Eingangsprofil die Art der Kommunikation, das Zurechtkommen in verschiedenen Kommunikationsumgebungen sowie eingeschränkte Gemeinschaftsaktivitäten erfasst. Darüber hinaus werden unter den Umweltfaktoren die persönliche und berufliche Lärm- und Geräuschumgebung, die Unterstützung durch (Familien‑)Angehörige sowie die Trageakzeptanz des Sprachprozessors erfragt und dokumentiert.

Erst wenn die Eingangsanalyse vollständig ausgefüllt ist, wird auf der Grundlage des Eingangsprofils ein individuell auf den Nutzer abgestimmter Trainingsplan durch den Übungsgenerator erstellt und dem zukünftigen Nutzer ein persönlicher Freischaltungscode per Mail übersandt.

#### Trainingsplan

**Figure 2:**
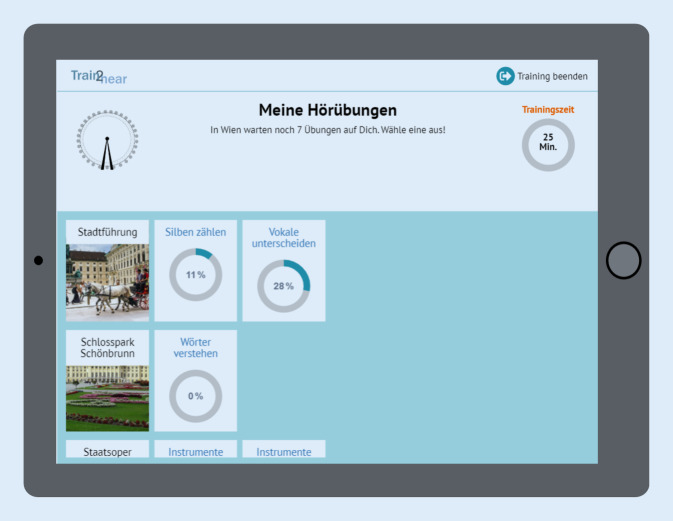

**Figure 3:**
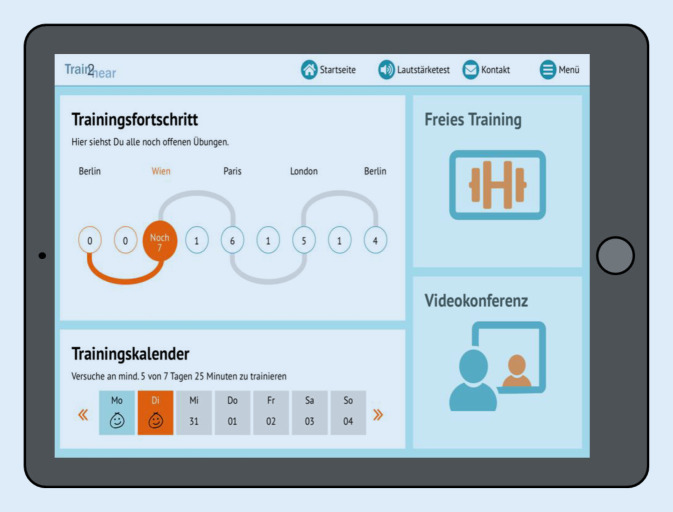

Eingebettet ist der *Trainingsplan* in eine fiktive Europareise von Berlin über Wien, Paris und London zurück nach Berlin, die der Nutzer in einer vorgeschriebenen Reihenfolge durchführen muss. Jeder Reiseort entspricht hierbei einer von fünf möglichen Schwierigkeitsstufen. Eine Weiterreise ist erst möglich, wenn eine festgelegte Anzahl an Übungen vom Patienten am jeweiligen Reiseort erfolgreich absolviert wurde (Abb. 2). In welcher Reihenfolge die Übungen an einem Reiseort durchgeführt werden, darf der Nutzer jedoch selbst bestimmen. Dies ist ein wichtiger Aspekt hinsichtlich des Motivationskonzeptes des Trainings. Während der fiktiven Fahrt im Zug („train2hear“) bzw. des fiktiven Flugs von einem zum nächsten Reiseort haben die Nutzer darüber hinaus die Möglichkeit, ein oder mehrere Spiele, z. B. ein Kreuzworträtsel oder ein Memory, zu absolvieren (Abb. 3). Ebenso steht den Nutzern ein freier Trainingsbereich zur Verfügung, in dem diese über die festgelegte Trainingszeit hinaus weitere Übungen absolvieren können.

### Elemente der Hörplattform

#### Adaptivität

Ein weiteres wichtiges Element der Plattform stellt die *Adaptivität *dar, der im Hinblick auf die Usability und Learnability eine große Bedeutung zukommt [12, 16]. Da die Software die Anzahl und Art der Fehler, die Bearbeitungsdauer, aber auch die Häufigkeit der Nutzung von Hilfestellungen kontinuierlich erfasst und unmittelbar an das administrative Backend weiterleitet, erfolgt eine automatische und dynamische Anpassung des Schwierigkeitsgrades der zu absolvierenden Übung.

**Figure 4:**
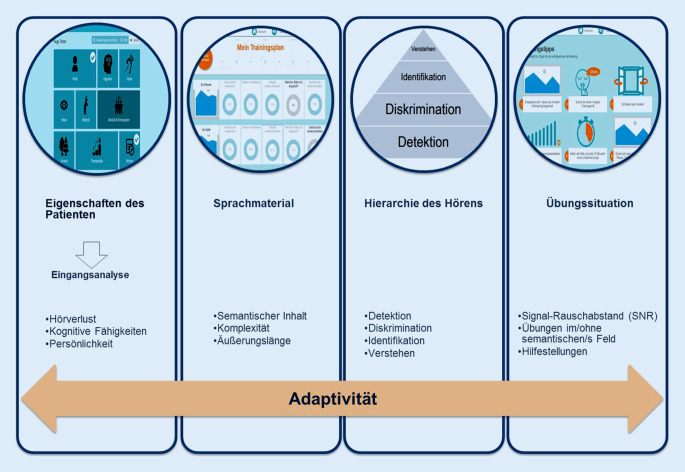

Adaptivität erfolgt dabei innerhalb einer Übung sowie zwischen den Übungen auf drei verschiedenen Ebenen (Ebene 1: Sprachmaterial, 2: Hörstufe und 3: Hörbedingungen) unter Berücksichtigung verschiedener Prinzipien, wie in (Abb. 4) dargestellt.

**Figure 5:**
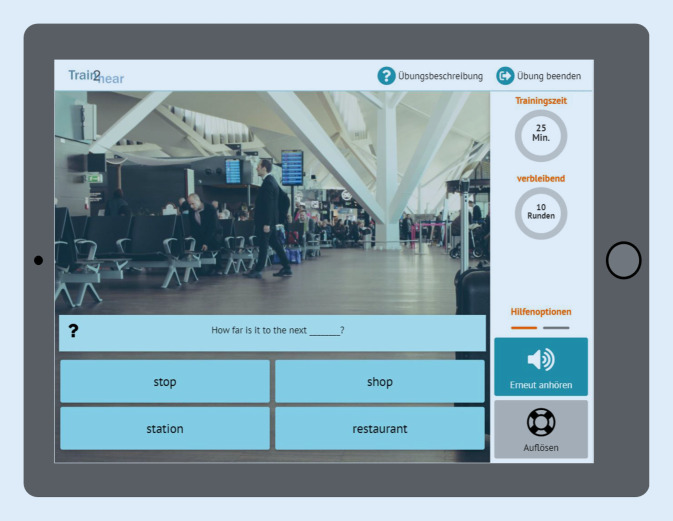

Auf der Ebene des sprachlichen Materials wird diese durch die Materialwahl (Silben, Wörter, Sätze, Texte), die sprachliche Komplexität und die zeitliche Länge des Materials (Wort‑, Satz- und Textlänge) erreicht (Abb. 5). Des Weiteren erfolgt Adaptivität mit zunehmendem Lernfortschritt durch den Einsatz immer höherer Hörstufen. Die dritte Ebene der Adaptivität bildet die Übungssituation. Die Schwierigkeit wird hier vor allem durch das Hinzufügen von permanenten Störgeräuschen mit einem immer geringer werdenden Signal-Rausch-Abstand verstärkt. Ebenso kann der Schwierigkeitsgrad durch die Auswahl des Settings („open set“ vs. „closed set“) beeinflusst werden.

Bei Schwierigkeiten stehen dem Nutzer unterschiedliche Hilfestellungen wie das Ausblenden des Störgeräusches oder die Wiederholung des zuvor gehörten Items zur Verfügung. Je nach Aufgabenstellung werden ein bzw. mehrere Prinzipien zur Erhöhung bzw. Vereinfachung des Schwierigkeitsgrades eingesetzt.

#### Feedback

**Figure 6:**
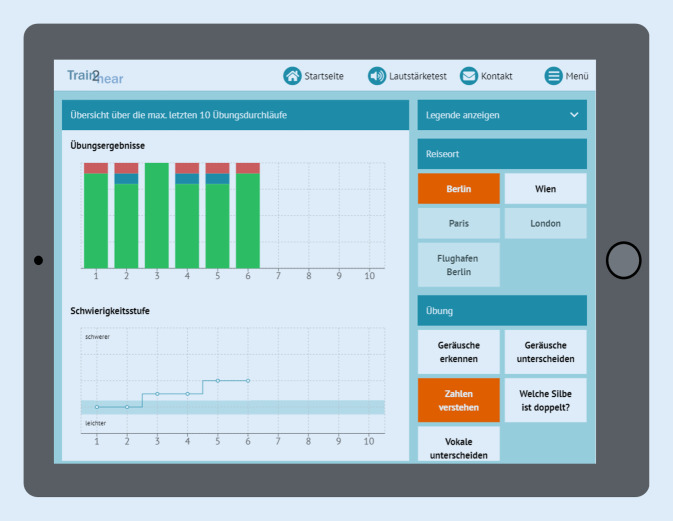

Das *Feedback*, das dem User während des Trainings gegeben wird, ist ergebnisbezogen, motivierend und korrektiv. So erhält der Nutzer nach jedem Übungsdurchlauf eine grafische Darstellung über die Anzahl der mit oder ohne Hilfestellung richtig und falsch gelösten Items zusätzlich zu einer kurzen schriftlichen Rückmeldung mit Bezug auf das Ziel der jeweiligen Übung. Alle Items des zuvor bearbeiteten Durchlaufs können nach Beendigung der Übung erneut vom Patienten angehört und mit der richtigen bzw. falschen Antwort verglichen werden. Daneben werden die Verlaufsdaten des Patienten auf einer ausführlichen Statistikseite, auf die sowohl der Patient als auch der Therapeut jederzeit Zugriff haben, dargestellt (Abb. 6). Auf der rechten Seite hat der Nutzer die Möglichkeit, die Übung nach Übungsort und Aufgabe auszuwählen. Zudem finden sich in der Therapeutenstatistik, die sich im therapeutischen Back-End befindet, auch erste Fehleranalysen für den einzelnen Patienten hinsichtlich der oben beschriebenen Merkmale und Ebenen.

#### Motivationskonzepte

**Figure 7:**
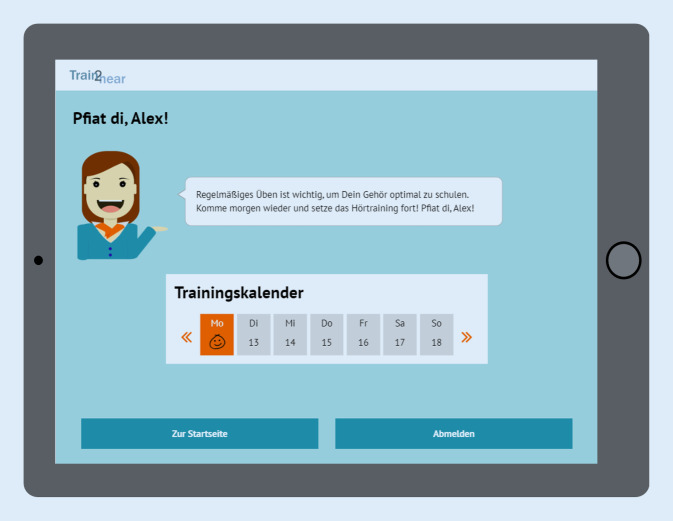

Verschiedene Elemente zur Steigerung der *Motivation* des Benutzers wurden integriert. Diese orientieren sich an der „Self Determination Theory“ von Ryan und Deci, welche die Selbstbestimmung, das Bedürfnis nach Kompetenz und Wirksamkeit sowie die soziale Eingebunden- und Zugehörigkeit als wesentliche motivierende Bausteine hervorhebt [39]. Neben spielerischen Elementen, wie der virtuellen Europareise, dem Erlangen von Trophäen und der Möglichkeit, Übungen in Form von Reisespielen zu absolvieren, enthält das Programm einen Avatar in der Person eines Zugführers, welcher den Patienten auf seiner Reise begleitet. Das Computerprogramm ist ein strukturiertes Übungsprogramm und gibt dem Nutzer vor, an mindestens fünf von sieben Tagen in der Woche jeweils 25 min zu üben, da regelmäßiges Üben für den Therapieerfolg besonders relevant ist. Die Zeitmessung findet im Hintergrund statt. Ein Trainingskalender gibt dem Nutzer ein visuelles Feedback zur Einhaltung der Trainingszeit. Ebenso wird der Nutzer durch den Zugbegleiter zu regelmäßigem Üben angeregt (Abb. 7).

## Diskussion

Die Nachfrage nach häuslichen computerbasierten Hörtrainingsprogrammen in der Rehabilitation nach einer Cochleaimplantation ist hoch. Eine eigene Bedarfsanalyse an 87 Cochlea-Implantierten zeigte, dass sich 75 % der Befragten ein häusliches Hörtraining über den Computer oder das Tablet gut vorstellen können. Die bisher verfügbaren Programme werden jedoch oft als eintönig bewertet. Von einem Computerprogramm erhoffen sich die User eine ausführliche Dokumentation der Trainingsfortschritte und ein differenziertes Feedback. An der derzeit noch gängigen Vor-Ort-Therapie in einem Rehabilitationszentrum werden hingegen die geringe Flexibilität und der hohe zeitliche und finanzielle Aufwand bemängelt [3].

Trotz des bestehenden Bedarfs gibt es bisher nur wenige deutschsprachige computerbasierte Trainingsprogramme, die auf erwachsene CI-Träger ausgerichtet sind [44]. Im Vergleich zum englischsprachigen Markt sind die deutschen Programme hinsichtlich ihrer Struktur, der wissenschaftlichen Evaluierung und den Inhalten her bislang nur wenig ausgereift.

Aktuell verfügbare Programme werden meist als freiwillige Ergänzung zur konventionellen Face-to-face-Therapie eingesetzt, mit dem Ziel, die Therapiefrequenz zu steigern und die Therapieinhalte zu vertiefen. Der Großteil der computerbasierten Hörtrainingsmodule wird dabei nicht von Therapeuten geleitet, vielmehr bestimmen die Nutzer Reihenfolge und Übungsauswahl selbst. Zudem existieren kaum Vorgaben zu Dauer und Häufigkeit des Trainings [44]. Eine Supervision des computerbasierten Trainings durch einen Therapeuten gibt es in keinem der bisher verfügbaren Programme. In einigen Programmen kann der Nutzer zwar selbständig eine Schwierigkeitsstufe auswählen, jedoch unabhängig davon, ob diese zum individuellen Leistungsniveau passt [44]. Ebenfalls verfügt keines der Programme über eine automatische Adaptivität der Aufgaben an das Leistungsniveau des Nutzers. Das von der Firma Med-EL (Innsbruck) konzipierte Training „Listen up!“ ermöglicht vor Trainingsbeginn eine Überprüfung des Sprachverständnisses, doch es erfolgt keine entsprechende adaptive Anpassung an die Testergebnisse. Ein effektives Hörtraining sollte sich jedoch am individuellen Leistungsniveau orientieren und bedarf daher – auch wenn es computerbasiert angeboten wird – einer adaptiven Gestaltung [12]. Einen strukturierten Trainingsplan, der als Grundlage für den Erfolg einer hörtherapeutischen Intervention gesehen wird, weisen die Programme „Online-Hörtraining-Verheyen“ (Jana Verheyen, Hamburg) und „Sensoton“ (Fa. Hoertraining-online.de, Hamburg) auf [25]. Ebenso geben diese beiden Programme eine Übungszeitempfehlung ab. Die Feedback- und Motivationskonzepte sind in allen verfügbaren Trainingsprogrammen meist nur sehr einfach gestaltet.

Ein therapeutisches Setting, das auf einem computerbasierten Programm beruht, ist nicht nur aus ökonomischer Sicht sinnvoll [35]. Die Veränderung des Settings von der Vor-Ort-Therapie hin zur Teletherapie kann, wie Studien gezeigt haben, zu einer gesteigerten Effizienz und Effektivität eines Trainings führen [43]. Außerdem besteht die Möglichkeit, Angehörige stärker in den Rehabilitationsprozess einzubinden. Dies hat, wie man bei Untersuchungen an Hörgeräteträgern nachweisen konnte, einen entscheidenden Einfluss auf das Outcome einer Hörrehabilitation [27, 32, 41].

Allerdings haben die Therapeuten häufig große Vorbehalte gegenüber einer Teletherapie, da sich die Therapeuten-Patienten-Beziehung, die als der tragender Part in der Sprach‑, Sprech- und Hörtherapie gilt, hierdurch stark wandelt [17, 33]. Eine stärkere Auseinandersetzung mit digitalen Therapieformen kann jedoch, wie Hines nachwies, diese Sichtweise langfristig ändern [22]. Mit der fortschreitenden Digitalisierung unserer Gesellschaft muss vermutlich in Zukunft auch die Rolle des Therapeuten neu überdacht werden. So wird dieser vermutlich zunehmend die Rolle eines Supervisors übernehmen und nur durch Videokonferenzen den persönlichen Austausch mit dem Patienten pflegen, wie in der hier vorgestellten train2hear-Plattform angedacht. Auch die Implementierung eines Avatars kann dazu beitragen, die Patienten während des computerbasierten Trainings unterstützend zu begleiten. Sicherlich bedarf es derzeit noch weiterer Forschungsarbeiten, um noch mehr zu analysieren, wie eine zufriedenstellende parasoziale Interaktion auch unter einem teletherapeutischen Training für den Nutzer gewährleistet werden kann [2, 26].

Eine sehr große Chance computerbasierter Trainingsprogramme liegt in der Möglichkeit, die Sprach‑, Sprech- und Hörtherapie, die derzeit meist noch wenig extern evidenzbasiert ist, weiter zu standardisieren und dadurch auch wissenschaftlich fundiert zu evaluieren [5]. Machbar ist dies durch die bislang noch ungeahnten und wenig genutzten Fähigkeiten digitaler Programme, eine Vielzahl an Daten zu speichern und bereitzustellen. Selbstlernende Algorithmen unter Anwendung von künstlicher Intelligenz (KI) bieten hier möglicherweise ganz neue therapeutische Ansätze. Hierdurch ist nicht nur eine genaue Analyse der zuvor definierten Fehler jedes einzelnen Users kontinuierlich während der gesamten Übungszeit möglich, sondern auch eine objektive Messung der Übungsfortschritte, Trainingszeiten oder aber der Reaktionszeiten, so ist der Vergleich im Rahmen von größeren Studien denkbar.

Das hier vorgestellte Hörtraining stellt unserer Kenntnis nach das erste dieser Art für CI-Träger im deutschsprachigen Raum dar, das eine umfassende Eingangsanalyse enthält, auf welche der Trainingsplan abgestimmt ist. Grundlage war das biopsychosoziale Modell der internationalen Weltgesundheitsorganisation (WHO ICF), das nicht nur die Funktionsstörung durch eine Hörstörung und die dadurch beeinträchtigen Aktivitäten und Teilhabe, sondern auch Umweltfaktoren und personenbezogene Faktoren berücksichtigt. Daneben passt sich das Programm durch die im Hintergrund ablaufende Analyse der erhobenen Daten kontinuierlich und automatisch im Schwierigkeitsgrad und bei der Auswahl der Übungen an den jeweiligen Leistungsstand des Einzelnen an. Wie Studien gezeigt haben, ist Adaptivität ein entscheidender Schlüssel zu einem effektiven Trainingsprogramm [15]. Indem das Schwierigkeitsniveau so gewählt werden kann, dass der Nutzer gefordert wird, ohne überfordert zu werden, soll ein im Hinblick auf das Lernen optimaler Flow entstehen [9, 34].

Ein kritischer Faktor für ein häusliches Training bleibt jedoch die Compliance des Patienten im Hinblick auf eine konsequente und regelmäßige eigenverantwortliche Durchführung desselben [7, 21]. Frühere Studien beschrieben eine große Varianz im Hinblick auf die Compliance von 30–100 % [20]. Zur Festigung der Adhärenz wurden daher unterschiedliche Strategien in der Trainingsplattform umgesetzt: So wurden festgelegte Trainingszeiten und die externe Kontrolle der täglichen Logins integriert, um die Häufigkeit der Nutzung des Programms durch den Nutzer selbst und auch extern evaluieren zu können. Auch wurden Möglichkeiten für Supervision und differenziertes Feedback, die entscheidend für die Abbrecherquote sind, in einer für Patienten und Therapeuten einsehbaren Statistik eingebaut [15, 21]. Derzeit gibt es, mit Ausnahme von einigen wenigen englischsprachigen Hörtrainingsprogrammen, kein Hörtraining, in dem eine enge Betreuung durch einen Therapeuten erfolgt.

Relevant für eine hohe Adhärenz ist auch die Motivation des Patienten. Henshaw konnte in einer Untersuchung bei Hörgeschädigten im Rahmen eines computerbasierten Phonemdiskriminationstrainings beobachten, dass eine fehlende Adhärenz auf eine mangelnde intrinsische Motivation zurückzuführen ist [21]. Laut der „Self Determination Theory“ von Ryan und Deci soll ein Gefühl von Individualität, Autonomie und Verbundenheit besonders motivierend sein [39]. Diese Elemente wurden in vielfältiger Weise im Programm „train2hear“ umgesetzt. So wird nicht nur das Trainingslevel durch die Adaptivität optimal auf den Patienten ausgerichtet, sodass dieser Erfolgserlebnisse zu verzeichnen hat. Ebenfalls wird das Feedback genutzt, um die Nutzer positiv zu bestärken. Durch die lokale und zeitliche Flexibilität des Trainings wird die Autonomie des Patienten hervorgehoben. Daneben hat der Nutzer die Möglichkeit, unabhängig vom festgelegten Trainingsplan in einem freien Trainingsbereich zu üben.

Um eine stärkere Compliance des Nutzers zu erzielen, wurden eine detaillierte Statistik über die Trainingserfolge und weiterführende Informationen in das Programm eingebettet. Dabei scheint vor allem die unmittelbare Rückmeldung, wie von Burk et al. nachgewiesen, von Bedeutung zu sein [6]. So erzielten normalhörende Probanden deutlichere Verbesserungen im Vergleich mit einer Kontrollgruppe im Wortverständnis, wenn das Hörtraining Feedback enthielt [6]. Mit Blick auf die User-Rückmeldung unserer Anwenderworkshops sollte daher langfristig der statistische Bereich noch weiter ausgebaut werden, ohne jedoch dabei den User mit zu viel Detailwissen zu überfordern. In Zukunft könnte die Plattform auch um einen Betroffenen-Chat und eine realistischere, alltagsbezogene virtuelle Realität ergänzt werden, um die soziale Integration des Betroffenen noch mehr zu stärken.

Wenngleich ein computerbasiertes Programm besonders für ältere Personen eine technische Herausforderung darstellen kann, unterschätzen Personen dieser Altersgruppe häufig ihre eigenen Kompetenzen [21]. Daher sollte ein computergestütztes Training vor der eigenständigen Nutzung zu Hause immer im persönlichen Kontakt im Therapiezentrum angeleitet werden. Auch wird man auf einen regelmäßigen Austausch mit den Therapeuten über eine Videokonferenz nicht verzichten können. Komorbiditäten, wie Sehstörungen und motorische Defizite, können die Nutzung besonders im höheren Lebensalter einschränken. Dementsprechend sollte die Entwicklung eines derartigen Programms die Nutzer- und Systemperspektive gleichermaßen berücksichtigen.

## Zusammenfassung

Das hier vorgestellte teletherapeutische computerbasierte Hörtraining stellt nach unserer Einschätzung eine große Chance sowohl für den einzelnen Hörgeschädigten als auch für die konzeptionelle Entwicklung der Hörrehabilitation im Allgemeinen dar. Derzeit steht noch die prospektive Evaluierung des vorgestellten Trainingsprogrammes an einem größeren Kollektiv aus. Multizentrische Studien sind in Zukunft sicherlich erforderlich, um die vielfältigen, noch offenen Fragen zur telemedizinischen Hörrehabilitation zu beantworten.

## Fazit für die Praxis

- Digitale Programme (in Form von Apps oder webbasiert) werden heute bereits als Ergänzung in der Hörrehabilitation nach Cochleaimplantation eingesetzt.
- Ein teletherapeutisches Hörtraining im Rahmen der CI(Cochleaimplantat)-Rehabilitation könnte eine ressourcenschonende und kostengünstige Alternative zur Face-to-face-Therapie darstellen.
- Die vorgestellte Plattform „train2hear“ stellt einen Prototypen eines möglichen computerbasierten Hörtrainings dar.
